# Demand for integrative medicine among women with breast and gynecological cancer: a multicenter cross-sectional study in Southern and Northern Germany

**DOI:** 10.1007/s00404-020-05880-0

**Published:** 2020-12-02

**Authors:** Donata Grimm, Sofia Mathes, Linn Woelber, Caroline Van Aken, Barbara Schmalfeldt, Volkmar Mueller, Marion Kiechle, Christine Brambs, Daniela Paepke

**Affiliations:** 1 Department of Gynecology and Gynecologic Oncology, University Medical Center Hamburg-Eppendorf, Martinistraße 52, 20246 Hamburg, Germany; 2 Department of Gynecology and Obstetrics, University Medical Center Schleswig-Holstein, Campus-Lübeck, Ratzeburger Allee 160, 23562 Lübeck, Germany; 3 Department of Gynecology and Obstetrics, Klinikum rechts der Isar, Technical University Munich, Ismaninger Straße 22, Munich, Germany

**Keywords:** Integrative medicine (IM), Complementary and alternative medicine (CAM), Gynecologic oncology, Breast cancer, Supportive care in cancer, Attitude of cancer patients, User behavior

## Abstract

**Purpose:**

The aim of this multicenter cross-sectional study was to analyze a cohort of breast (BC) and gynecological cancer (GC) patients regarding their interest in, perception of and demand for integrative therapeutic health approaches.

**Methods:**

BC and GC patients were surveyed at their first integrative clinic visit using validated standardized questionnaires. Treatment goals and potential differences between the two groups were evaluated.

**Results:**

340 patients (272 BC, 68 GC) participated in the study. The overall interest in IM was 95.3% and correlated with older age, recent chemotherapy, and higher education. A total of 89.4% were using integrative methods at the time of enrolment, primarily exercise therapy (57.5%), and vitamin supplementation (51.4%). The major short-term goal of the BC patients was a side-effects reduction of conventional therapy (70.4%); the major long-term goal was the delay of a potential tumor progression (69.3%). In the GC group, major short-term and long-term goals were slowing tumor progression (73.1% and 79.1%) and prolonging survival (70.1% and 80.6%). GC patients were significantly more impaired by the side-effects of conventional treatment than BC patients [pain (*p* = 0.006), obstipation (< 0.005)].

**Conclusion:**

Our data demonstrate a high overall interest in and use of IM in BC and GC patients. This supports the need for specialized IM counseling and the implementation of integrative treatments into conventional oncological treatment regimes in both patient groups. Primary tumor site, cancer diagnosis, treatment phase, and side effects had a relevant impact on the demand for IM in our study population.

## Introduction

Over the past decade “Complementary and Alternative Medicine” (CAM) as well as “Integrative Oncology” have become increasingly popular as part of a holistic treatment approach in gynecologic oncology. This is partly due to the fact that advances in conventional cancer treatments have led to a prolonged life expectancy and a more pronounced interest in short-term and long-term quality of life (QoL).

According to the National Center for Complementary and Integrative Health (NCCIH), CAM is the popular name for non-mainstream health care practices that are not part of the standard medical treatment and are used to support or replace conventional medical therapies (complementary versus alternative, respectively) [1–3]. Integrative medicine (IM) differs from CAM because it focuses on a more holistic approach while being supported by evidence, with emphasis on the therapeutic relationship between the practitioner and patients. IM provides a framework in which to combine conventional and complementary treatment approaches and neither rejects conventional therapies nor accepts alternative approaches uncritically [3–7].

There has been a marked increase in the demand for IM in the oncological patient population over the past years [4]. IM is being used by up to 43% of all cancer patients worldwide (e.g., 50−70% in Germany, 45−49% in Australia, and up to 95% in the US) [8−17]. The use is particularly pronounced among breast cancer patients (BC) who apply IM strategies in up to 90% of cases [18]. Owing to patients’ varying cultural backgrounds, resulting in differences in their acceptance of IM, there is great heterogeneity in the implementation of IM around the world [4]. In Germany, 40−70% of all patients with gynecologic malignancies and approximately 50−75% of BC patients use IM concomitant to their conventional therapy [19–21]. Mistletoe was the most commonly used (77%) complementary treatment [20].

A significant correlation between an interest in IM and a younger age (< 60 years) and the absence of metastases at the time of diagnosis has been described in BC patients [13, 22−27]. In addition, IM use has been shown to be more common in female patients, is associated with a higher economic status, a higher level of knowledge of their disease, and a higher educational level [13, 22–26]. IM along with conventional treatment or after the completion of the primary therapy is commonly a primarily supportive treatment with the purpose of mitigating physical issues from the cancer itself or the treatment to support physical and psychological well being [27, 28]. Moreover, IM methods are often applied with the goal to improve QoL, relieve chemotherapy (CTX)-induced symptoms, boost the immune system, and even increase survival [29, 30].

Although there is data on the prevalence of IM use for oncological patients, there is little data regarding IM for those patients treated in highly specialized academic oncological centers (comprehensive cancer centers), and information is still limited on the potential impact of counseling for different cancer patient subgroups (e.g., BC and GC).

The aim of this multicenter cross-sectional study was to evaluate the interest in, use of and demand for IM in patients with BC and GC in the setting of a comprehensive cancer center. In addition, we wanted to identify individual treatment goals that patients sought to achieve using IM, as well as the frequency of the applied IM methods. Lastly, we wanted to elucidate the association between patient and therapy-related characteristics and adverse effects and to gather information on two different specialized integrative consultancy services in the south and north of Germany.

## Materials and methods

Over a period of 20 months, the multicenter nonsponsored cross-sectional study of the Department of Gynecology and Obstetrics, Technical University Munich (TUM), Klinikum rechts der Isar and the Department of Gynecology and Gynecologic Oncology at the University Medical Center Hamburg-Eppendorf (UKE) prospectively enrolled 340 patients with gynecologic malignancies (TUM *n* = 229, UKE *n* = 111) aged 23−84 who sought counseling in a specialized IM service. Both comprehensive cancer centers had implemented IM as part of clinical routine in a standardized form years ago. The physicians who counsel with regard to IM are specialists in gynecological oncology and members of the “Arbeitsgemeinschaft Gynäkologische Onkologie Kommission Integrative Medicine” (AG IMed) who are certified in naturopathic and anthroposophic medicine as well as nutrition sciences.

Patients interested in IM were asked to complete a questionnaire called the “AG IMed questionnaire”. This is a validated, standardized questionnaire developed by the AG IMed research group, and designed to obtain basic information on the use of IM among gynecologic cancer patients [2]. Its 43 questions cover general information (including personal and demographic data, information on the disease, previous, and current treatments, co-medications, social environment), lifestyle (including exercise and diet), the use of complementary and alternative therapies, a person’s physical and mental state (including complaints, QoL) and patientsʼ treatment goals (including wishes and expectations). Specific individual IM treatment recommendations were offered based on the data gathered from the questionnaire as well as patients’ medical records in addition to standard cancer therapies. Biologically based medicine, nutritional and lifestyle regulations, exercise therapy as well as hydrotherapy and physiotherapy, the classic disciplines of traditional European naturopathic medicines, were included in the treatment plans.

Inclusion criteria were the diagnosis of BC or any GC, a minimum age of 18 years, a follow-up at either UKE or TUM, the ability to understand the questions as well as the grasp of written and spoken German. All patients sought medical advice regarding IM in addition to the conventional treatment of their carcinomas.

The study protocol was designed in accordance with the Declaration of Helsinki, and ethical approval was obtained from the respective ethics committees (reference number 255_16B).

### Statistics

Descriptive statistics, such as absolute and relative frequencies as well as means and standard deviations were generated to determine the prevalence and patterns of CAM use, the demand for IM approaches, the differences in the types of cancer, the frequency of metastases, the current treatment, symptomatic complaints and the treatment goals the patients hoped to achieve. An one-way ANOVA and chi-squared test were conducted for hypothesis testing on potential associations between the interest in IM and the application of CAM, as well as sociodemographic characteristics. A potential association between the type of cancer and the two comprehensive cancer centers were analyzed using chi-square analysis for the comparison of absolute and relative frequencies and Fisher´s exact test (chi square with Yates correction for small sample size). Patients with missing values were excluded from the analysis of the corresponding variables. The level of significance was set at *p* < 0.05. The data management and statistical analyses were performed using the statistical software SPSS, Version 20 (IBM Corp., Armonk, New York, USA).

## Results

340 women (TUM *n* = 229, UKE *n* = 111) with a median age of 53 (range 23−84) were included in the study. The distribution of the different types of carcinomas is displayed in Table 1. In the following analysis, all gynecological cancer types, such as ovarian-, cervical-, endometrial- and vulvar cancer are summarized as the gynecological cancer group (GCG). At the time of enrolment, more than a quarter of the patients (27.7%, *n* = 94/339) had metastasized disease (UKE 39.6%, *n* = 44/111; TUM 21.9%, *n* = 50/228, *p* = 0.001) (Table 1).

**Table 1:** Patient characteristics (*n* = 340) showing absolute numbers, percentages, and means

Characteristics	No./total (%)
Number of patients	340 (100%)
Age (years)	
Mean ± SD	52.8 ± 12.3
Median (range)	53 (23−84)
Age ≤ 40	58/340 (17.1%)
Age 41−60	192/340 (56.5%)
Age > 60	90/340 (26.5%)
Cancer type in both centers (UKE^1^ and TUM^2^)	
Breast cancer (BC)	272/340 (80.0%)
Gynecological cancer^3^ (GC)	68/340 (20.0%)
Ovarian cancer	51/340 (15.0%)
Cervical cancer	9/340 (2.6%)
Endometrial cancer	6/340 (1.8%)
Vulvar cancer	2/340 (0.6%)
Children per patient	
Mean (SD)	1.3 ± 1.1
Median (range)	1 (0−6)
Not known	3/340 (0.9%)
Postmenopausal	270/338 (79.9%)
Body mass index (BMI)	
Underweight (BMI < 18.5 kg/m^2^)	12/338 (3.6%)
Normal weight (BMI 18.5−25.0 kg/m^2^)	214/338 (63.3%)
Overweight (BMI > 25.0 kg/m^2^)	112/338 (33.1%)
Unknown^4^	2/340 (0.6%)
Family status	
Married patients or patients in a solid relationship	253/337 (75.1%)
Unmarried patients or patients with no solid relationship	84/337 (24.9%)
Not known	3/340 (0.9%)
Education	
No school qualification	2/338 (0.6%)
Secondary school	141/338 (41.7%)
High school graduation	63/338 (18.6%)
College/university degree	132/338 (39.1%)
Not known	2/340 (0.6%)
Smoker	
No	192/337 (57.0%)
Yes	145/337 (43.0%)
Active smokers	20/337 (5.9%)
History of smoking	125/337 (37.1%)
Not known	3/340 (0.9%)
Alcohol	
Never	175/338 (51.8%)
1−2 times per week	129/338 (38.2%)
3−6 times per week	29/338 (8.6%)
Every day	5/338 (1.5%)
Not known	2/340 (0.6%)
Exercise	
Never	64/328 (19.5%)
Once a week	116/328 (35.4%)
2−4 times a week	112/328 (34.1%)
> 4 times a week	36/328 (11.0%)
Not known	12/340 (3.5%)
Diabetes	
No	312/334 (93.4%)
Yes	22/334 (6.6%)
Not known	6/340 (1.8%)
Family history of any cancer	
Yes	240/339 (70.8%)
No	99/339 (29.2%)
Not known	1/340 (0.3%)
Treatment phase at the time of presentation	
Neoadjuvant	91/340 (26.8%)
Adjuvant	127/340 (37.4%)
Palliative	113/340 (33.2%)
Watchful waiting (at patient´s request)	1/340 (0.3%)
Surgery (at patient´s request)	5/340 (1.5%)
Only CAM^5^ (at patient´s request)	3/340 (0.9%)
Metastatic disease at the time of diagnosis (both centers)	
Metastatic disease	94/339 (27.7%)
No metastatic disease	245/339 (72.3%)
Not known	1/340 (0.3%)
*Type of therapy*	
Radiotherapy	
Current radiotherapy	24/337 (7.1%)
Recent radiotherapy	101/337 (30.0%)
No radiotherapy	212/337 (62.9%)
Not known	3/340 (0.9%)
Chemotherapy (CTX)	
Current CTX	209/338 (61.8%)
Recent CTX	62/338 (18.3%)
No CTX	67/338 (19.8%)
Not known	2/340 (0.6%)
Antihormonal treatment	
Current antihormonal treatment	65/333 (19.5%)
Recent antihormonal treatment	32/333 (9.6%)
No antihormonal treatment	236/333 (70.9%)
Not known	7/340 (2.1%)
Targeted therapy	
Current targeted therapy	88/327 (26.9%)
Recent targeted therapy	25/327 (7.6%)
No targeted therapy	214/327 (65.4%)
Not known	13/340 (3.8%)
Bisphosphonate therapy	
Current bisphosphonate therapy	31/322 (9.6%)
Recent bisphosphonate therapy	10/322 (3.1%)
No bisphosphonate therapy	281/322 (87.3%)
Not known	18/340 (5.3%)
Participation in clinical trials	
Current trial participation	86/328 (26.2%)
Recent trial participation	25/328 (7.6%)
No trial participation	217/328 (66.2%)
Not known	12/340 (3.5%)

Multiple responses for cancer treatments were allowed

^1^UKE: University Medical Center Hamburg-Eppendorf

^2^TUM: Technical University Munich

^3^Gynecological cancer includes patients with ovarian-, cervical-, endometrial-, and vulvar cancer

^4^Two patients refused to disclose their weight

^5^CAM: Complementary and alternative medicine

Within the questionnaire, the general interest in IM was assessed, and patients with and without interest in IM were compared by univariate analysis. A total of 95.3% of patients reported a general interest in IM. There was a higher interest in IM in patients aged > 60 years than in younger patients (Table 2). Further, a total of 46.5% of patients with metastatic disease indicated they had become interested in IM since the initial diagnosis, as opposed to 37.6% with localized disease. Patients with a recent CTX were significantly more interested in IM compared to patients who had not undergone CTX (46.7%, *n* = 28/60 vs. 28.3%, *n* = 17/60, *p* = 0.040). There was a significant association between a higher degree of education and a higher interest in IM (*p* = 0.021) (Table 2).

**Table 2:** Association between the interest in integrative medicine (IM) and epidemiological/treatment characteristics based on the time since diagnosis

Interest in integrative medicine (IM)					
Characteristics	IM interest		No IM interest		
	∑ = 306/321 (95.3%)				
	Interest prior to diagnosis	Interest after diagnosis	Unknown		
All	177/321 (55.1%)	129/321(40.2%)	15/321 (4.7%)	19/340 (5.6%)	*p* value
Age					
Age ≤ 40 years	29/54 (53.7%)	22/54 (40.7%)	3/54 (5.6%)		0.410
Age 41−60 years	109/185 (58.9%)	67/185 (36.2%)	9/185 (4.9%)		
Age > 60 years	39/82 (47.6%)	40/82 (48.8%)	3/82 (3.7%)		
Body mass index (BMI)^1^					
Underweight (BMI < 18.5 kg/m^2^)	4/12 (33.3%)	8/12 (66.7%)	0/12 (0.0%)		0.248
Normal weight (BMI 18.5−25.0 kg/m^2^)	121/206 (58.7%)	76/206 (36.9%)	9/206 (4.4%)		
Overweight (BMI > 25.0 kg/m^2^)	51/101 (50.5%)	44/101 (43.6%)	6/101 (5.9%)		
Education^2^					
Secondary school	60/130 (46.2%)	60/130 (46.2%)	10/130 (7.7%)		**0.021**
High school graduation	37/59 (62.7%)	19/59 (32.2%)	3/59 (5.1%)		
College/university degree	79/129 (61.2%)	48/129 (37.2%)	2/129 (1.6%)		
Cancer type					
Breast cancer	148/259 (57.1%)	101/259 (39.0%)	10/259 (3.9%)		0.181
Gynecological cancer	29/62 (46.8%)	28/62 (45.2%)	5/62 (8.1%)		
Metastatic disease at the time of diagnosis^3^					
Metastasis	42/86 (48.8%)	40/86 (46.5%)	4/86 (4.7%)		0.328
No metastasis	135/234 (57.7%)	88/234 (37.6%)	11/234 (4.7%)		
Treatment phase at the time of diagnosis ^4^					
Adjuvant	76/125 (60.8%)	43/125 (34.4%)	6/125 (4.8%)		0.262
Neoadjuvant	48/89 (53.9%)	38/89 (42.7%)	3/89 (3.4%)		
Palliative	47/101 (46.5%)	48/101 (47.5%)	6/101 (5.9%)		
Chemotherapy (CTX)					
Current CTX	103/199 (51.8%)	83/199 (41.7%)	13/199 (6.5%)		**0.040**
Recent CTX	32/60 (53.3%)	28/60 (46.7%)	0/60 (0.0%)		
No CTX	41/60 (68.3%)	17/60 (28.3%)	2/60 (3.3%)		

Significant results in bold print; the results were analyzed using Fisher´s exact test (chi-square with Yates correction for small sample size) for the comparison of absolute and relative frequencies

^1^Two patients refused to disclose their weight

^2^Two patients had no school-leaving qualifications and were excluded from the statistical evaluation due to the small number of cases

^3^One patient had not undergone imaging for staging yet

^4^The categories ‘watchful waiting (at patient´s request)’, ‘surgery (at patient´s request)’, and ‘only CAM (at patient´s request)’ were excluded from the statistical analysis due to the small number of cases (*n* = 9)

A total of 63.8% of patients had previously (before study enrolment) applied some IM methods and 89.2% patients were using some IM method at the time of the study enrolment (27.7% of patients had metastatic disease, 61.8% patients were currently treated with CTX, 18.3% had recently (before study enrolment) undergone a CTX while 19.8% had never received CTX). The *use* of any integrative method increased from the time prior to the time of study enrolment across all subgroups before > after diagnosis (*recent CTX*: 73.8% (45/61) > 91.8% (56/61), *current CTX*: 60.5% (124/205) > 86.8% (178/205), *no-CTX*: 65.2% (43/66) > 95.5% (63/66), *metastasis:* 67.4% (62/92) > 89.1% (82/92)). Patients who had recently undergone a CTX applied more methods of IM at the time of enrolment than patients without a prior CTX or currently under CTX (5.92 ± 4.15 vs. 5.21 ± 4.67 vs. 3.51 ± 3.12; (*p* < 0.001)). The amount of different IM methods increased with higher educational levels.

**Fig. 1:**
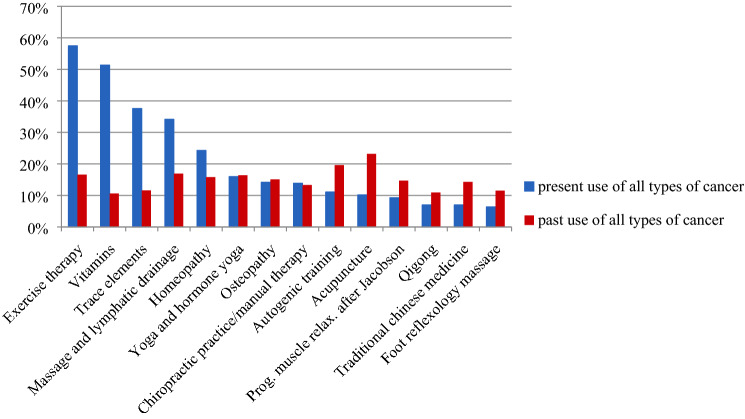
Frequent integrative methods sorted by highest increase in use of the method comparing presence and past. Values are calculated as relative frequencies within the total cohort of breast cancer (BC) and gynecological cancer (GC) patients

The difference between the use of different IM methods at present and in the past is depicted in Fig. 1.

Both in the BC group (BCG) and the GCG, the reduction of potential side effects of the conventional therapies, the stabilization of body, soul, and spirit, as well as active participation in the treatment of their cancer were the most important short-term goals. The delay of a potential progression of disease, an attempt to prolong survival as well as the stabilization of body, soul, and spirit were major long-term goals (Table 3). A possible association between patient- and therapy-related characteristics and adverse effects was also evaluated (Table 4).

**Table 3:** Treatment goals of breast cancer and gynecological cancer patients with regard to integrative medicine

	Breast cancer *n* = 272 (100%)	Gynecological cancer^1^ *n* = 68 (100%)	*p* value
Missing value	2/272 (0.7%)	1/68 (1.5%)	
Relief of cancer-associated symptoms			
Short-term goal	**105/270 (38.9%)**	**37/67 (55.2%)**	**0.015**
Long-term goal	**83/270 (30.7%)**	**31/67 (46.3%)**	**0.016**
Reduction of side effects of conventional therapy			
Short-term goal	190/270 (70.4%)	45/67 (67.2%)	0.609
Long-term goal	133/270 (49.3%)	33/67 (49.3%)	0.999
Improvement of disease-related quality of life			
Short-term goal	165/270 (61.1%)	44/67 (65.7%)	0.491
Long-term goal	140/270 (51.9%)	41/67 (61.2%)	0.170
Improvement of coping with the disease			
Short-term goal	139/270 (51.5%)	31/67 (46.3%)	0.445
Long-term goal	123/270 (45.6%)	31/67 (46.3%)	0.916
Stabilization of body, soul, and spirit			
Short-term goal	185/270 (68.5%)	44/67 (65.7%)	0.655
Long-term goal	179/270 (66.3%)	38/67 (56.7%)	0.143
Active participation in treatment of the disease			
Short-term goal	176/270 (65.2%)	43/67 (64.2%)	0.877
Long-term goal	139/270 (51.5%)	39/67 (58.2%)	0.323
Slowing of progression of disease			
Short-term goal	**157/270 (58.1%)**	**49/67 (73.1%)**	**0.024**
Long-term goal	187/270 (69.3%)	53/67 (79.1%)	0.111
Prolonging survival time			
Short-term goal	**149/270 (55.2%)**	**47/67 (70.1%)**	**0.026**
Long-term goal	**172/270 (63.7%)**	**54/67 (80.6%)**	**0.008**

Absolute numbers and percentages are shown

Significant results in bold print

Multiple responses were allowed, p < 0.05 (significant results in bold print); the results were analyzed using chi-square analysis for the comparison of absolute and relative frequencies

^1^Gynecological cancer includes patients with ovarian-, cervical-, endometrial-, and vulvar cancer

**Table 4:** Association of patient and therapy-related characteristics and adverse effects

Age no/total (%)					
	Age ≤ 40 years	Age 41−60 years	Age > 60 years	All	*p* value
Adverse effects					
Reduced cogitation	20/57 (35.1%)	60/190 (31.6%)	17/89 (19.1%)	97/336 (28.9%)	0.053
Fatigue	31/57 (54.4%)	103/190 (54.2%)	56/89 (62.9%)	190/336 (56.5%)	0.367
Pain	22/57 (38.6%)	69/190 (36.3%)	45/89 (50.6%)	136/336 (40.5%)	0.074
Climacteric symptoms	9/57 (15.8%)	31/190 (16.3%)	10/89 (11.2%)	50/336 (14.9%)	0.527
Diarrhea	9/57 (15.8%)	25/190 (13.2%)	8/89 (9.0%)	42/336 (12.5%)	0.440
Obstipation	12/57 (21.1%)	27/190 (14.2%)	18/89 (20.2%)	57/336 (17.0%)	0.306
Depression	14/57 (24.6%)	45/190 (23.7%)	29/89 (32.6%)	88/336 (26.2%)	0.276
Impaired sexual activity	11/57 (19.3%)	36/190 (18.9%)	9/89 (10.1%)	56/336 (16.7%)	0.153
Missing value				4/340 (1.2%)	

Body Mass Index no/total (%)						
	Underweight^1^	Normalweight^2^		Overweight^3^	All	*p* value
Adverse effects						
Reduced cogitation	2/12 (16.7%)	60/213 (28.2%)		34/109 (31.2%)	96/334 (28.7%)	0.605
Fatigue	5/12 (41.7%)	116/213 (54.5%)		68/109 (62.4%)	189/334 (56.6%)	0.226
Pain	5/12 (41.7%)	80/213 (37.6%)		49/109 (45.0%)	134/334 (40.1%)	0.444
Climacteric symptoms	1/12 (8.3%)	33/213 (15.5%)		16/109 (14.7%)	50/334 (15.0%)	0.961
Diarrhea	1/12 (8.3%)	28/213 (13.1%)		13/109 (11.9%)	42/334 (12.6%)	0.952
Obstipation	5/12 (41.7%)	33/213 (15.5%)		17/109 (15.6%)	55/334 (16.5%)	0.080
Depression	2/12 (16.7%)	51/213 (23.9%)		35/109 (32.1%)	88/334 (26.3%)	0.221
Impaired sexual activity	1/12 (8.3%)	37/213 (17.4%)		18/109 (16.5%)	56/334 (16.8%)	0.858
Missing value					6/340 (1.8%)	

Education no/total (%)						
	Secondary modern school	High-school graduation		Post-graduated	All	*P* value
Adverse effects						
Reduced cogitation	38/140 (27.1%)	22/62 (35.5%)		35/130 (26.9%)	95/332 (28.6%)	0.414
Fatigue	79/140 (56.4%)	31/62 (50.0%)		76/130 (58.5%)	186/332 (56.0%)	0.539
Pain	73/140 (52.1%)	24/62 (38.7%)		37/130 (28.5%)	134/332 (40.4%)	** < 0.001**
Climacteric symptoms	17/140 (12.1%)	14/62 (22.6%)		19/130 (14.6%)	50/332 (15.1%)	0.158
Diarrhea	19/140 (13.6%)	10/62 (16.1%)		12/130 (9.2%)	41/332 (12.3%)	0.336
Obstipation	26/140 (18.6%)	10/62 (16.1%)		20/130 (15.4%)	56/332 (16.9%)	0.772
Depression	41/140 (29.3%)	10/62 (16.1%)		35/130 (26.9%)	86/332 (25.9%)	0.136
Impaired sexual activity	24/140 (17.1%)	11/62 (17.7%)		20/130 (15.4%)	55/332 (16.6%)	0.893
Missing value					8/340 (2.4%)	

Cancer type no/total (%)						
	Breast cancer		Gynecological cancer		All	*p* value
Adverse effects						
Reduced cogitation	75/269 (27.9%)		22/67 (32.8%)		97/336 (28.9%)	0.423
Fatigue	149/269 (55.4%)		41/67 (61.2%)		190/336 (56.5%)	0.391
Pain	99/269 (36.8%)		37/67 (55.2%)		136/336 (40.5%)	**0.006**
Climacteric symptoms	40/269 (14.9%)		10/67 (14.9%)		50/336 (14.9%)	0.991
Diarrhea	34/269 (12.6%)		8/67 (11.9%)		42/336 (12.5%)	0.877
Obstipation	38/269 (14.1%)		19/67 (28.4%)		57/336 (17.0%)	**0.005**
Depression	65/269 (24.2%)		23/67 (34.3%)		88/336 (26.2%)	0.090
Impaired sexual activity	42/269 (15.6%)		14/67 (20.9%)		56/336 (16.7%)	0.299
Missing value					4/340 (1.2%)	

Metastases no/total (%)						
	Metastases		No metastases		All	P value
Adverse effects						
Reduced cogitation	21/93 (22.6%)		76/242 (31.4%)		97/335 (29.0%)	0.111
Fatigue	58/93 (62.4%)		131/242 (54.1%)		189/335 (56.4%)	0.174
Pain	49/93 (52.7%)		87/242 (36.0%)		136/335 (40.6%)	**0.005**
Climacteric symptoms	14/93 (15.1%)		36/242 (14.9%)		50/335 (14.9%)	0.967
Diarrhea	13/93 (14.0%)		29/242 (12.0%)		42/335 (12.5%)	0.621
Obstipation	17/93 (18.3%)		39/242 (16.1%)		56/335 (16.7%)	0.635
Depression	28/93 (30.1%)		60/242 (24.8%)		88/335 (26.3%)	0.322
Impaired sexual activity	17/93 (18.3%)		39/242 (16.1%)		56/335 (16.7%)	0.635
Missing value					5/340 (1.5%)	

Therapy no/total (%)						
	Adjuvant	Neoadjuvant		Palliative	All	P value
Adverse effects						
Reduced cogitation	40/126 (31.7%)	26/90 (28.9%)		29/112 (25.9%)	95/328 (29.0%)	0.610
Fatigue	55/126 (43.7%)	57/90 (63.3%)		74/112 (66.1%)	186/328 (56.7%)	**0.001**
Pain	52/126 (41.3%)	23/90 (25.6%)		58/112 (51.8%)	133/328 (40.5%)	**0.001**
Climacteric symptoms	24/126 (19.0%)	7/90 (7.8%)		17/112 (15.2%)	48/328 (14.6%)	0.068
Diarrhea	12/126 (9.5%)	12/90 (13.3%)		16/112 (14.3%)	40/328 (12.2%)	0.495
Obstipation	21/126 (16.7%)	11/90 (12.2%)		24/112 (21.4%)	56/328 (17.1%)	0.222
Depression	32/126 (25.4%)	18/90 (20.0%)		36/112 (32.1%)	86/328 (26.2%)	0.144
Impaired sexual activity	23/126 (18.3%)	9/90 (10.0%)		21/112 (18.8%)	53/328 (16.2%)	0.175
Missing value					12/340 (3.5%)	

Chemotherapy no/total (%)						
	Current CTX	Recent CTX		No CTX	All	P value
Adverse effects						
Reduced cogitation	63/207 (30.4%)	20/61 (32.8%)		14/66 (21.2%)	97/334 (29.0%)	0.276
Fatigue	127/207 (61.4%)	35/61 (57.4%)		27/66 (40.9%)	189/334 (56.6%)	**0.014**
Pain	78/207 (37.7%)	37/61 (60.7%)		20/66 (30.3%)	135/334 (40.4%)	**0.001**
Climacteric symptoms	22/207 (10.6%)	14/61 (23.0%)		13/66 (19.7%)	49/334 (14.7%)	**0.025**
Diarrhea	29/207 (14.0%)	8/61 (13.1%)		5/66 (7.6%)	42/334 (12.6%)	0.386
Obstipation	47/207 (22.7%)	4/61 (6.6%)		6/66 (9.1%)	57/334 (17.1%)	**0.002**
Depression	55/207 (26.6%)	15/61 (24.6%)		18/66 (27.3%)	88/334 (26.3%)	0.936
Impaired sexual activity	32/207 (15.5%)	17/61 (27.9%)		6/66 (9.1%)	55/334 (16.5%)	**0.014**
Missing value					6/340 (1.8%)	

Multiple responses were allowed, p < 0.05 (significant results in bold print); the results were analyzed using chi-square analysis for the comparison of absolute and relative frequencies and Fisher´s exact test (Chi square with Yates correction for small sample size)

^1^Underweight: body mass index < 18.5 kg/m^2^

^2^Normalweight: body mass index 18.5−25.0 kg/m^2^

^3^Overweight: body mass index > 25.0 kg/m^2^

### Comparison of breast and gynecological cancer patients

**Fig. 2:**
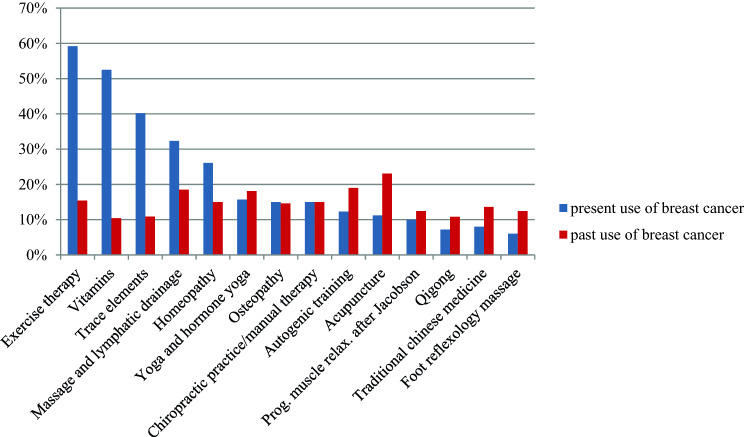
Frequency of present and past used integrative methods in breast cancer (BC) patients

**Fig. 3:**
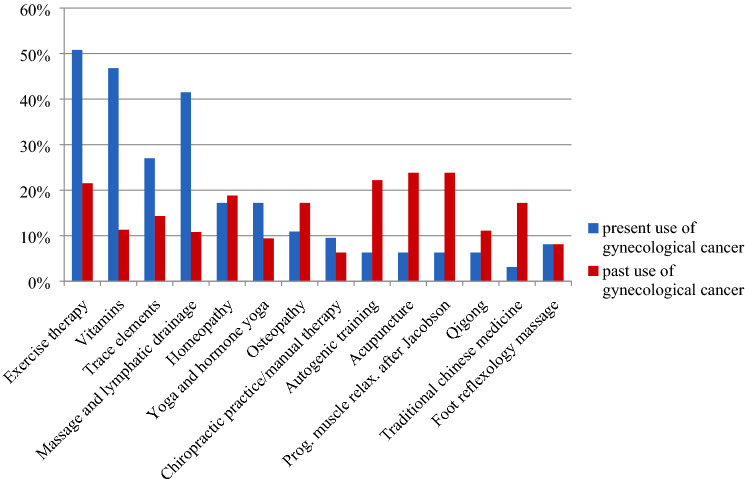
Frequency of present and past used integrative methods in gynecological cancer (GC) patients

At the time of enrolment 249 patients (96.1%) with BC and 57 GC patients (91.9%) indicated an interest in IM and a total of 238 (89.1%) BC patients and 60 (89.6%) GC patients were using some IM method. The methods used in both groups are shown in Figs. 2 and 3. Table 3 summarizes the goals for IM use in BC versus GC. The delay of a progression of disease as a short-term goal was significantly more pronounced in patients with GC than with BC (73.1%, *n* = 49/67 in the GCG vs. 58.1%, *n* = 157/270 in the BCG; *p* = 0.024). In addition, significantly more patients with GC reported the prolongation of survival time (short-term 70.1%, *n* = 47/67 in the GCG vs. 55.2%, *n* = 149/270 in the BCG (*p* = 0.026); long-term: 80.6%, *n* = 54/67 in the GCG vs. 63.7%, *n* = 172/270 in the BCG (*p* = 0.008)) and a reduction of cancer-associated symptoms (short-term 55.2%, *n* = 37/67 in the GCG vs. 38.9%, *n* = 105/270 in the BCG (*p* = 0.015); long-term: 46.3%, *n* = 31/67 in the GCG vs. 30.7%, *n* = 83/270 in the BCG (*p* = 0.016)) as a short-term or long-term goal.

Chemotherapy-associated side effects, such as reduced cognition, fatigue, pain, menopausal symptoms, diarrhea, obstipation, depression, and reduced sexual activity were also analyzed in BC and GC patients (Table 4). More patients undergoing CTX suffered from fatigue (current CTX: *n* = 127/207 (61.4%) vs. recent CTX: *n* = 35/61 (57.4%) vs. no CTX: *n* = 27/66 (40.9%), *p* = 0.014) and obstipation (current CTX: *n* = 47/207 (22.7%) vs. recent CTX: *n* = 4/61 (6.6%) vs. no CTX: *n* = 6/66 (9.1%), *p* = 0.002) than patients after recent CTX or without CTX. Patients with the recent CTX had more pain (recent CTX: *n* = 37/61 (60.7%) vs. current CTX: *n* = 78/207 (37.7%) vs. no CTX: *n* = 20/66 (30.3%), *p* = 0.001), climacteric symptoms (recent CTX: *n* = 14/61 (23.0%) vs. current CTX: *n* = 22/207 (10.6%) vs. no CTX: *n* = 13/66 (19.7%), *p* = 0.025) and impaired sexual activity (recent CTX: *n* = 17/61 (27.9%) vs. current CTX: *n* = 32/207 (15.5%) vs. no CTX: *n* = 6/66 (9.1%), *p* = 0.014) than patients with current CTX or without CTX. Patients with palliative therapy were more affected by fatigue (palliative: *n* = 74/112 (66.1%) vs. adjuvant: *n* = 55/126 (43.7%) vs. neoadjuvant: *n* = 57/90 (63.3%), *p* = 0.001) and pain (palliative: *n* = 58/112 (51.8%) vs. adjuvant: *n* = 52/126 (41.3%) vs. neoadjuvant: *n* = 23/90 (25.6, %), *p* = 0.001) than patients treated in a curative setting.

## Discussion

In line with previous research, our data reflect the recent trend towards an increased interest in IM (> 90%) among BC- and GC patients. Likewise, there was comparably high use of IM at the time of study enrolment (89.1% in the BCG and 89.6% in the GCG), and a mean number of four IM methods were being applied (BCG 4.45 SD ± 3.98 and GCG 3.54 SD ± 2.82).

When compared with the existing evidence, this demonstrates an even higher use of IM; a number of studies may have underestimated the high prevalence of IM among women with BC and GC [21, 25, 27, 31–33]. IM use has increased in the recent years and shows strong regional variations, ranging from 40 to 70% in Germany to 94.7% in Turkey [9, 19, 20, 34, 35]. Molassiotis et al. [13] conducted a cross-sectional study and included 282 BC patients from 14 countries in Europe. Forty-four percent (44.7%) of BC patients used IM after their cancer diagnosis (e.g., Italy 73.1%, Czech Republic 58.8%, Switzerland 48.6%, Greece 14.8%). In the United States in 2002, 49.6% of GC patients used some IM method after cancer was diagnosed [29].

With an estimated incidence of almost two million BC cases and nearly 300,000 new cases of OC in 2018 around the globe, the interest in and frequent use of IM applies to a large number of patients [36]. Many studies have tried to identify a typical profile of IM users according to sociodemographic or disease-related data. Looking at characteristics for IM users, the data shows that the use of IM is more common among females, younger patients (mostly < 60 years), and patients with a higher level of education and nonmetastatic disease [16, 20, 21, 29, 31, 37–39]. However, other studies failed to reflect this association [37, 40, 41]. Within our study, patients > 60 years of age (48.8%), well educated (high school and college > secondary modern school), with a recent or current CTX as well as those suffering from metastatic disease showed the highest interest in IM (Table 2). This is in contrast to the findings of Fremd et al. who recently analyzed the interest in IM among German BC patients [21]. In their cohort, IM interest correlated with being younger (mean age 51.7 years) and the absence of metastases at the time of diagnosis [21]. The discrepancy in age might be due to infrastructure or informational flow within the two clinics. The distribution of flyers as well as active communication about IM and easy access to counseling services directly implemented in conventional therapy in both departments might lower the threshold for making additional appointments, especially for older people. A lack of infrastructure and/or information might hinder elderly patients from putting their interest into practice.

There is immense variation in preferred IM practices between studies from different countries; practices are often chosen based on a specific cultural background [19, 42–44]. In our study, the IM methods most frequently used in the BCG as well the GCG were exercise therapy (BCG 59.2%, GCG 50.8%) and vitamins (BCG 52.5%, GCG 46.8%). Patients with BC were more likely to use trace elements (40.2%), whereas GC patients favored massage and lymphatic drainage (41.5%) (Figs. 2 and 3). Therapies used in the past had often been based on courses (e.g., progressive muscle relaxation, acupuncture, autogenic training, etc.) and could be used again in the further course of the disease. The former data suggests that patients treated at cancer centers with an associated integrative medicine service seemed to be well informed about evidence-based integrative strategies, such as exercise therapy and healthy nutrition.

The current medical field of exercise interventions in BC is enormous. Large observational studies on BC patients show an inverse relationship between physical activity (before and after diagnosis) and overall mortality, BC-specific mortality, and BC events (progression, relapse, and new disease) [45, 46]. Exercise seems to have a positive influence on certain cancers- and treatment-related side effects, QoL, recurrence, and survival in BC patients.

In OC patients, physical activity seems just as important, although evidence is scarce, particularly on long-term outcomes. OC continues to have a poor prognosis, with a 5-year survival rate of approximately 43% across all stages (RKI 2016). Faced with a poor prognosis, stressful treatments and a high likelihood of recurrence, women with OC confront significant physical and psychological morbidities that are likely to have negative influence on their health-related QoL. In our cohort, GC patients are more impaired in their QoL than patients with BC, reaching statistical significance in the category pain (*p* = 0.006) and obstipation (*p* = < 0.005). Symptom-related research in patients with a malignancy has shown that physical activity is associated with improvement concerning physiological complaints, such as pain, peripheral neuropathy, and fatigue as well as in the psychological symptoms of anxiety and depression [47–52]. However, the influence of physical activity on survival and QoL in patients diagnosed with OC remains unexplored. The majority of OC survivors showed an interest in participating in physical activity programs [50], and smaller pilot studies have proven the feasibility of such programs during and after CTX in the setting of primary and recurrent OC [52–55]. There is evidence that physical activity during treatment and follow-up can improve cachexia by reducing the tumor's adverse effects on muscle metabolism, insulin sensitivity, and levels of inflammation [53–56]. Consistent with these findings, a lack of physical activity prior to the diagnosis of OC has been shown to be associated with lower overall survival in 6,806 patients [57]. The results of the BENITA trial, a prospective randomized controlled trial that is evaluating exercise and nutrition interventions for OC patients during and after first-line CTX, are due for publication in 2021.

According to our data, GC patients seem to benefit from physical activity as well as massage and lymphatic drainage (Fig. 3). Besides physical activity, the use of vitamin supplementation was widespread in both patient groups, perhaps due to disease-based or treatment-related reduced nutritional intake and/or involuntary weight loss. It is currently widely acknowledged that nutritional interventions can improve calorie intake, maintain or improve body weight, improve body composition, prevent sarcopenia, and reduce treatment toxicity in oncological patients [58].

Within our cohort, 89.4% of BC and GC patients used IM in addition to conventional therapy (19.5% alongside antihormonal treatment, 61.8% with CTX, 7.1% with radiotherapy, and 26.9% with targeted therapies).

Reducing the side effects of conventional oncological therapies, stabilizing body, soul, and spirit, and active treatment participation were the most important short-term goals of BC patients (Table 3). The major long-term goals were a reduction in the progression of disease, a prolongation of survival, and a stabilization of body, soul and spirit (Table 3). This is similar to the results from Hack et al. [59]. Interestingly, Hack et al. reported the slowing of tumor progression as the most important treatment goal in BC patients (85.3%). This goal was less commonly stated in the current evaluation (69.3% of our BCG), while the reduction of side effects from conventional therapy (80%) and active participation in treatment of the disease (74.7%) were given more importance [59]. In contrast to our data, Hack et al. did not differentiate between short-term and long-term goals. A higher rate of patients in a palliative setting in our cohort (33% vs 20%) might explain these differing treatment goals.

The data regarding the goals of GC patients in integrative oncology are scarce. Within our cohort, prolonging survival, slowing the progression of disease, and improving disease-related QoL were major short-term and long-term goals in GC patients. Interestingly, neither a delay of disease progression nor an improvement in survival was a short-term or a long-term goal in 30−40% of all patients (Table 3). To date, not much is known about the complex factors that make patients with cancer choose QoL rather than gain of life expectancy. Decision making in cancer treatment is difficult because there are multiple features to consider aside from purely medical aspects [60]. Likewise, the compromises a patient is willing to make can vary greatly, depending on many factors that include patient age, family dynamics, social structures and patients' likely survival, and baseline QoL [60]. At any time of treatment, clinicians should offer extra time for patients to discuss their personal goals over time to align personal and treatment goals with regularity and to ensure adequate support. However, financially sustainable structures are not available now and many clinicians regret not having enough time for individual patient treatment. This lack of provider-led communication poses a potential safety risk as it may lead to adverse interactions between IM methods and oncological treatments as well as noncompliance [59]).

A survey conducted in 11 European countries among women with BC demonstrated that almost half of the BC patients (45%) apply IM methods, starting at the time of diagnosis, and that most are satisfied with them. Only 6.5% of these women reported no benefit from IM [39]. A recent German survey revealed that 59% of BC or GC patients would have been interested in receiving information on IM from their physicians, and more than half of the patients (54%) expressed their wish for an implementation of IM into routine oncological care. Many patients (38%) reported that their primary sources of information regarding IM had been family members and friends and not their medical providers [26].

This again underlines the importance of specialized integrative services at comprehensive cancer centers where patients can be counseled on safe and effective IM methods to help alleviate treatment-based and disease-related side-effects while keeping potential interactions with chemotherapies and other conventional treatments in mind and creating a larger body of evidence.

Trained health-care professionals, a standardized setting, good communications skills, time, and an open discussion on IM issues are key to protect patients from an inappropriate and potentially dangerous use of IM. Oncologists should proactively communicate information on IM and explain possible supportive treatment options at the same time they make patients aware of the potential harm of some of these therapies.

The results of the current study have some limitations. First, our survey data are prone to recall bias since it is based on the self-reporting. Patients presenting to our associated integrative medicine service were generally interested in IM; thereby, introducing an element of selection bias. Secondly, regardless of their stage of disease, BC and GC patients must be considered a rather heterogeneous group. Understandably, different stages and phases of disease may result in different demands for integrative medicine support services. Thirdly, some of the studies quoted include mind/body therapies, others solely evaluated "biological therapies". Overall, the wide range of any generous definition of IM and CAM may result in a study overestimating the number of IM users [61].

To our knowledge, this is the first bicentric observational study analyzing patients with both BC and GC who seek counseling in IM at two large comprehensive cancer centers in Germany. Patients of all ages and tumor stages are strongly interested in IM as a supplement to their conventional oncological treatment. In contrast to other studies, this survey underlines that patients older than 60 years are also interested in IM. The results show that nutritional counseling as well as physiotherapeutic approaches such as physical activity, massage, and lymph drainage should be routinely offered to patients and financially supported as part of their cancer treatment. Further psychological counseling as well as mind–body approaches should be offered within a comprehensive oncological treatment approach.

## Conclusion

The alleviation of physical symptoms and the desire to improve health behaviors is of paramount importance to BC and GC patients in regards to their supportive care needs. BC and GC patients place a special focus on IM methods to achieve these goals. The primary tumor site, BC versus ovarian cancer, treatment phase and side effects had a relevant impact on the patients` demand for IM in the current study. Therefore, it is crucial to incorporate IM counseling into the conventional oncological therapy. Since comprehensive cancer centers spearhead oncological research and innovations, these centers need to ensure a financially sustainable integration of IM into the oncological treatment and follow-up. In addition, evidence-informed IM needs to expand beyond the walls of academic medical centers in order to ensure access to IM for patients from diverse socioeconomic backgrounds in an oncological setting.

## Data Availability

Detailed data are available by e-mailing Donata Grimm (d.grimm@uke.de and donatakatharina.grimm@uksh.de) and Daniela Paepke (Daniela.Paepke@mri.tum.de).

## References

[1] Baum M, Ernst E, Lejeune S, Horneber M (2006). Role of complementary and alternative medicine in the care of patients with breast cancer: report of the European Society of Mastology (EUSOMA) Workshop, Florence, Italy, December 2004. Eur J Cancer.

[2] Hack CC, Huttner NB, Fasching PA, Beckmann MW (2015). Development and validation of a standardized questionnaire and standardized diary for use in integrative medicine consultations in gynecologic oncology. Geburtshilfe Frauenheilkd.

[3] 5. NCCIH. Complementary a, or integrative health: What’s in a name? https://nccih.nih.gov/health/integrative-health-integrative. Accessed July 24, 2019.

[4] Kalder M, Muller T, Fischer D, Muller A, Bader W, Beckmann MW (2016). A review of integrative medicine in gynaecological oncology. Geburtshilfe Frauenheilkd.

[5] Leis AM, Weeks LC, Verhoef MJ (2008). Principles to guide integrative oncology and the development of an evidence base. Curr Oncol.

[6] Seely DM, Weeks LC, Young S (2012). A systematic review of integrative oncology programs. Curr Oncol.

[7] http://exploreim.ucla.edu/health-care/east-meets-west-how-integrative-medicine-is-changing-health-care/ Assesed July.

[8] Yun H, Sun L, Mao JJ (2017) growth of integrative medicine at leading cancer centers between 2009 and 2016: a systematic analysis of nci-designated comprehensive cancer center websites. J Natl Cancer Inst Monogr 2017(52). 10.1093/jncimonographs/lgx004 PMC6061227 29140485

[9] Horneber M, Bueschel G, Dennert G, Less D, Ritter E, Zwahlen M (2012). How many cancer patients use complementary and alternative medicine: a systematic review and metaanalysis. Integr Cancer Ther.

[10] Boon H, Stewart M, Kennard MA, Gray R, Sawka C, Brown JB (2000). Use of complementary/alternative medicine by breast cancer survivors in Ontario: prevalence and perceptions. J Clin Oncol.

[11] Boon HS, Olatunde F, Zick SM (2007). Trends in complementary/alternative medicine use by breast cancer survivors: comparing survey data from 1998 and 2005. BMC Women's Health.

[12] Lettner S, Kessel KA, Combs SE (2017). Complementary and alternative medicine in radiation oncology: survey of patients' attitudes. Strahlentherapie und Onkologie.

[13] Molassiotis A, Fernandez-Ortega P, Pud D, Ozden G, Scott JA, Panteli V (2005). Use of complementary and alternative medicine in cancer patients: a European survey. Ann Oncol.

[14] Huebner J, Prott FJ, Micke O, Muecke R, Senf B, Dennert G (2014). Online survey of cancer patients on complementary and alternative medicine. Oncol Res Treat.

[15] Rausch SM, Winegardner F, Kruk KM, Phatak V, Wahner-Roedler DL, Bauer B (2011). Complementary and alternative medicine: use and disclosure in radiation oncology community practice. Sup Care Cancer.

[16] Wilkinson JM, Stevens MJ (2014). Use of complementary and alternative medical therapies (CAM) by patients attending a regional comprehensive cancer care centre. J Complement Integr Med.

[17] Smith PJ, Clavarino AM, Long JE, Anstey CM, Steadman KJ (2016). Complementary and alternative medicine use by patients receiving curative-intent chemotherapy. Asia Pacific J Clin Oncol.

[18] Conrad AC, Muenstedt K, Micke O, Prott FJ, Muecke R, Huebner J (2014). Attitudes of members of the German Society for Palliative Medicine toward complementary and alternative medicine for cancer patients. J Cancer Res Clin Oncol.

[19] Munstedt K, Kirsch K, Milch W, Sachsse S, Vahrson H (1996). Unconventional cancer therapy–survey of patients with gynaecological malignancy. Arch Gynecol Obstet.

[20] Fasching PA, Thiel F, Nicolaisen-Murmann K, Rauh C, Engel J, Lux MP (2007). Association of complementary methods with quality of life and life satisfaction in patients with gynecologic and breast malignancies. Sup Care Cancer.

[21] Fremd C, Hack CC, Schneeweiss A, Rauch G, Wallwiener D, Brucker SY (2017). Use of complementary and integrative medicine among German breast cancer patients: predictors and implications for patient care within the PRAEGNANT study network. Arch Gynecol Obstet.

[22] Vapiwala N, Mick R, Hampshire MK, Metz JM, DeNittis AS (2006). Patient initiation of complementary and alternative medical therapies (CAM) following cancer diagnosis. Cancer J.

[23] Burstein HJ, Gelber S, Guadagnoli E, Weeks JC (1999). Use of alternative medicine by women with early-stage breast cancer. New Engl J Med.

[24] Cassileth BR, Deng G (2004). Complementary and alternative therapies for cancer. Oncologist.

[25] Ernst E, Cassileth BR (1998). The prevalence of complementary/alternative medicine in cancer: a systematic review. Cancer.

[26] Schuerger N, Klein E, Hapfelmeier A, Kiechle M, Brambs C, Paepke D (2019). Evaluating the demand for integrative medicine practices in breast and gynecological cancer patients. Breast Care (Basel).

[27] Drozdoff L, Klein E, Kiechle M, Paepke D (2018). Use of biologically-based complementary medicine in breast and gynecological cancer patients during systemic therapy. BMC Complement Alternat Med.

[28] Akpunar D, Bebis H, Yavan T (2015). Use of complementary and alternative medicine in patients with gynecologic cancer: a systematic review. Asian Pacific J Cancer Prevent.

[29] Swisher EM, Cohn DE, Goff BA, Parham J, Herzog TJ, Rader JS (2002). Use of complementary and alternative medicine among women with gynecologic cancers. Gynecol Oncol.

[30] von Gruenigen VE, Frasure HE, Jenison EL, Hopkins MP, Gil KM (2006). Longitudinal assessment of quality of life and lifestyle in newly diagnosed ovarian cancer patients: the roles of surgery and chemotherapy. Gynecol Oncol.

[31] Wanchai A, Armer JM, Stewart BR (2010). Complementary and alternative medicine use among women with breast cancer: a systematic review. Clin J Oncol Nurs.

[32] Paul M, Davey B, Senf B, Stoll C, Munstedt K, Mucke R (2013). Patients with advanced cancer and their usage of complementary and alternative medicine. J Cancer Res Clin Oncol.

[33] Schurger N, Klein E, Hapfelmeier A, Kiechle M, Paepke D (2018). Demand for integrative medicine among women in pregnancy and childbed: a German survey on patients' needs. BMC Complement Alternat Med.

[34] Downer SM, Cody MM, McCluskey P, Wilson PD, Arnott SJ, Lister TA (1994). Pursuit and practice of complementary therapies by cancer patients receiving conventional treatment. BMJ.

[35] Molassiotis A, Scott JA, Kearney N, Pud D, Magri M, Selvekerova S (2006). Complementary and alternative medicine use in breast cancer patients in Europe. Sup Care Cancer.

[36] Bray F, Ferlay J, Soerjomataram I, Siegel RL, Torre LA, Jemal A (2018). Global cancer statistics 2018: GLOBOCAN estimates of incidence and mortality worldwide for 36 cancers in 185 countries. CA Cancer J Clin.

[37] Huebner J, Muenstedt K, Prott FJ, Stoll C, Micke O, Buentzel J (2014). Online survey of patients with breast cancer on complementary and alternative medicine. Breast Care (Basel).

[38] Judson PL, Abdallah R, Xiong Y, Ebbert J, Lancaster JM (2017). Complementary and alternative medicine use in individuals presenting for care at a comprehensive cancer center. Integr Cancer Ther.

[39] Molassiotis A, Browall M, Milovics L, Panteli V, Patiraki E, Fernandez-Ortega P (2006). Complementary and alternative medicine use in patients with gynecological cancers in Europe. Int J Gynecol Cancer.

[40] Molassiotis A, Ozden G, Platin N, Scott JA, Pud D, Fernandez-Ortega P (2006). Complementary and alternative medicine use in patients with head and neck cancers in Europe. Eur J Cancer Care.

[41] Zhang Y, Leach MJ, Hall H, Sundberg T, Ward L, Sibbritt D (2015). Differences between male and female consumers of complementary and alternative medicine in a national US population: a secondary analysis of 2012 NIHS data. Evid Based Complement Alternat Med.

[42] Ernst E, Resch KL, White AR (1995). Complementary medicine. What physicians think of it: a meta-analysis. Arch Internal Med.

[43] Cutshall S, Derscheid D, Miers AG, Ruegg S, Schroeder BJ, Tucker S (2010). Knowledge, attitudes, and use of complementary and alternative therapies among clinical nurse specialists in an academic medical center. Clin Nurse Spec CNS.

[44] McKay DJ, Bentley JR, Grimshaw RN (2005). Complementary and alternative medicine in gynaecologic oncology. J Obstet Gynaecol.

[45] Friedenreich CM, Neilson HK, Farris MS, Courneya KS (2016). Physical activity and cancer outcomes: a precision medicine approach. Clin Cancer Res.

[46] Kondrup J, Rasmussen HH, Hamberg O, Stanga Z (2003). Nutritional risk screening (NRS 2002): a new method based on an analysis of controlled clinical trials. Clin Nutr.

[47] Mustian KM, Sprod LK, Palesh OG, Peppone LJ, Janelsins MC, Mohile SG (2009). Exercise for the management of side effects and quality of life among cancer survivors. Curr Sports Med Rep.

[48] Mustian KM, Peppone LJ, Palesh OG, Janelsins MC, Mohile SG, Purnell JQ (2009). Exercise and cancer-related fatigue. US Oncol.

[49] Nicholas PK, Kemppainen JK, Canaval GE, Corless IB, Sefcik EF, Nokes KM (2007). Symptom management and self-care for peripheral neuropathy in HIV/AIDS. AIDS Care.

[50] Stevinson C, Faught W, Steed H, Tonkin K, Ladha AB, Vallance JK (2007). Associations between physical activity and quality of life in ovarian cancer survivors. Gynecol Oncol.

[51] Stevinson C, Steed H, Faught W, Tonkin K, Vallance JK, Ladha AB (2009). Physical activity in ovarian cancer survivors: associations with fatigue, sleep, and psychosocial functioning. Int J Gynecol Cancer.

[52] Moonsammy SH, Guglietti CL, Santa Mina D, Ferguson S, Kuk JL, Urowitz S (2013). A pilot study of an exercise & cognitive behavioral therapy intervention for epithelial ovarian cancer patients. J Ovar Res.

[53] Grande AJ, Silva V, Maddocks M (2015). Exercise for cancer cachexia in adults: executive summary of a Cochrane Collaboration systematic review. J Cach Sarcop Mus.

[54] Maddocks M, Murton AJ, Wilcock A (2012). Therapeutic exercise in cancer cachexia. Crit Rev Oncog.

[55] Maddocks M, Jones LW, Wilcock A (2013). Immunological and hormonal effects of exercise: implications for cancer cachexia. Curr Opin Sup Palliat Care.

[56] Bronger H, Hederich P, Hapfelmeier A, Metz S, Noel PB, Kiechle M (2017). Sarcopenia in advanced serous ovarian cancer. Int J Gynecol Cancer.

[57] Cannioto RA, LaMonte MJ, Kelemen LE, Risch HA, Eng KH, Minlikeeva AN (2016). Recreational physical inactivity and mortality in women with invasive epithelial ovarian cancer: evidence from the Ovarian Cancer Association Consortium. Br J Cancer.

[58] Ravasco P (2019). Nutrition in cancer patients. J Clin Med.

[59] Hack CC, Antoniadis S, Hackl J, Langemann H, Schwitulla J, Fasching PA (2018). Breast cancer patients' satisfaction with individual therapy goals and treatment in a standardized integrative medicine consultancy service. Arch Gynecol Obstet.

[60] Shrestha A, Martin C, Burton M, Walters S, Collins K, Wyld L (2019). Quality of life versus length of life considerations in cancer patients: a systematic literature review. Psycho Oncol.

[61] Keene MR, Heslop IM, Sabesan SS, Glass BD (2019). Complementary and alternative medicine use in cancer: a systematic review. Complement Ther Clin Pract.

